# The implementation of Journeying through Dementia: Strategies to run a successful pragmatic multicenter trial of a complex intervention

**DOI:** 10.1002/brb3.2436

**Published:** 2021-11-13

**Authors:** Katherine Berry, Jessica Wright, Kirsty Sprange, Cindy Cooper, Rebecca Courtney‐Walker, Gail Mountain

**Affiliations:** ^1^ Manchester Academic Health Science Centre The University of Manchester Manchester England; ^2^ Greater Manchester Mental Health NHS Foundation Trust The University of Manchester Manchester England; ^3^ Sheffield Clinical Trials Research Unit School of Health and Related Research University of Sheffield Sheffield England; ^4^ Nottingham Clinical Trials Unit Faculty of Medicine University of Nottingham Nottingham England; ^5^ Sheffield Clinical Trials Research Unit School of Health and Related Research University of Sheffield Sheffield England; ^6^ Cumbria Northumberland Tyne and Wear NHS Foundation Trust St. Nicholas Hospital Newcastle upon Tyne England; ^7^ The Centre for Applied Dementia Studies, Faculty of Health Studies University of Bradford Bradford England

**Keywords:** complex intervention, dementia, implementation, pragmatic trial

## Abstract

**Objective:**

A key challenge in delivering pragmatic trials of complex interventions is effective implementation within the study period and beyond. We describe a trial of an intervention to improve quality of life in mild dementia (Journeying through Dementia), describe some of the challenges raised in terms of implementation, and illustrate the methods used to ensure effective implementation.

**Method:**

The intervention was delivered by staff within local services and supervised by more experienced clinicians within those services in order to test the intervention in real‐world settings and establish the potential for future embedding into practice. Researchers delivered training sessions for all facilitators and supervisors, met at regular intervals with intervention supervisors, and provided feedback on summaries of intervention sessions created by facilitators. We conducted a thematic analysis of the content of meetings and written correspondence between the researchers and intervention supervisors regarding implementation issues.

**Results:**

Key themes relating to difficulties with implementation were: staff absences and staff leaving posts; participant lack of engagement with intervention; difficulties with delivery of supervision; difficult group dynamics; lack of time to deliver the intervention; and lack of adherence to the intervention and its ethos.

**Conclusion:**

We provide guidance for researchers involved in the trialing of other complex interventions in how these challenges might be overcome. These include: recruiting additional staff to deliver the intervention; having clear protocols in place for managing staff absences; using supervision to problem solve participant attendance at intervention sessions and difficult group dynamics; monitoring staff engagement in supervision and addressing problems with engagement with staff and managers when this occurs; giving staff ring‐fenced time to deliver the intervention and engage in supervision; and regular monitoring and feedback in relation to the content of the intervention to ensure that it is consistent with ethos and content of the intervention manual.

## Abstract 2

In delivering trials of complex interventions for dementia, it is important to provide training on all aspects of the intervention to staff, to have clear and detailed manuals, and to monitor adherence within the context of supervision with oversight from experienced clinicians within the research team.

## 1 INTRODUCTION

A complex intervention is a programme of interconnected components, which may be implemented in a variety of ways to address problems in health and social care settings (Craig et al., 2008). New complex interventions should be evaluated through randomized controlled trials (RCTs) (Craig et al., 2008). RCTs are either explanatory or pragmatic. Explanatory trials measure the benefit of an intervention using a homogenous, well‐defined sample of participants with highly trained interventionists. Pragmatic trials measure effectiveness—the benefit of the intervention for routine practice (Tosh et al., 2011).

One key problem in the delivery of pragmatic trials of complex interventions is poor implementation of intervention models (Ditcher et al., 2017; Sturkenboom et al., 2016). Implementation refers to reach (the proportion of participants receiving the intervention); fidelity (whether the intervention is delivered as planned); and dose delivered and received (the amount of intervention delivered and the extent to which participants responded to it) (Linnan & Steckler, 2002). Complex interventions have scope for variation in delivery so are vulnerable to components not being implemented as intended (Carroll et al., 2007). This is problematic as the level of implementation is a key moderator of outcomes (Carroll et al., 2007). Researchers often attempt to optimize implementation through obtaining “buy in” from key stakeholders, the use of manuals, training and supervision and monitoring and feedback (Gearing et al., 2011; Powell et al., 2013).

In this paper, we describe our experiences as researchers in overseeing the delivery of a complex intervention within a pragmatic RCT. In describing our experiences, we aim to highlight to other researchers the challenges that can present in implementing and evaluating complex interventions within the context of pragmatic RCTs. We also aim to use our collective experiences of successful and less successful intervention implementation both within this trial and other trials to describe how some of these challenges might be overcome.

## 2 METHOD

### 2.1 Summary of trial and intervention

The Journeying through Dementia intervention was designed to promote independence, self‐efficacy, and continued participation in life by people with mild dementia. It involved 12 weekly, 2‐h facilitated groups with 8−12 participants with dementia delivered in a community venue, as well as four one‐to‐one sessions ideally with the same facilitator for individual goal setting. The intervention was designed for delivery by two Band 4 staff NHS Agenda for Change members (either healthcare support workers or assistant psychologists who were not registered health or social care professionals) and supervised by a minimum Band 7 staff member NHS Agenda for Change (either a senior nurse or other allied healthcare professional).

The mannualized intervention contained a menu of topics, which were identified through consultation with people with dementia (Mountain & Craig, 2012) and explored within a feasibility study (Sprange et al, 2015). Topics included understanding dementia, relationships, physical and mental well‐being, daily living, skill development, and planning for the future. Each group session involved the same structure, which included information giving, discussion, and practical activity with an essential component being enactment of activities in the community with support from each other and the facilitators. The content of the one‐to‐one sessions was guided by the participant's choice but also involved enactment of activities in the home and/or community. The first one‐to‐one session with each participant took place before the commencement of the groups. This provided an opportunity for the facilitator to meet the participant and discuss the practicalities of attending groups and any concerns.

Facilitators were asked to document all intervention sessions on a proforma provided by the trial team and post these to the participants before the next session, thereby providing a record and reminder of what had taken place. One‐to‐one session records were intended to be written in collaboration with participants. Facilitators were told that all documents should be written in accordance with DEEP (Dementia Empowerment and Engagement Project) guidance for best practice in providing documentation for people with dementia (e.g., first person, accessible language, etc.) (DEEP, 2013).

A large, multicenter RCT was conducted to understand how effective Journeying through Dementia was in improving well‐being, self‐management abilities, and independence of people with mild dementia and how much the intervention cost compared to treatment as usual (Wright et al., 2019). The trial involved 13 sites and 480 participants diagnosed with dementia. There were 28 groups across the sites involving 69 facilitators and 21 supervisors. The trial included a fidelity substudy to determine if the intervention was delivered as planned and help understand the study outcomes, as opposed to using findings to inform and improve delivery during the trial (Sprange et al., 2021a). The trial also included a qualitative study to help contextual findings (Sprange et al., 2021b).

### 2.2 Procedures to support successful implementation of the intervention

Facilitators received training prior to delivering the intervention. Once intervention delivery commenced, facilitators all received weekly supervision for at least an hour from a more senior professional from their place of work who had also received training in the intervention model and the role of supervision in the trial. Facilitators and those supervising them all maintained records of the nature and duration of each supervision session using trial proformas. A supervision protocol was provided to guide supervision and included the importance of a mix of individual and group supervision with facilitators (see Sprange et al., 2021 supplementary material).

In order to circumvent the challenges associated with delivering a new intervention, which neither supervisors nor facilitators had prior experience of, members of the research team delivered a mixture of individual and group supervision to the supervisors via monthly face‐to‐face or phone meetings. These members of the research team were experienced clinical psychologists who undertook the same training as facilitators, but also had extensive previous experience of both delivering and supervising psychological therapies as part of RCTs. As a further measure to ensure that the intervention was being delivered as intended, facilitators were asked to photocopy records and other documents relating to the intervention and send them to the research team for review. Sites were initially requested to send the records at the end of the 12‐week intervention period. However, to improve intervention fidelity on an on‐going basis, half way through the study, site interventionists were asked to return records every three weeks. Generic cross‐site feedback on deviations and consequent further guidance was provided by email communication to all sites actively delivering the intervention. Personalized feedback was not given to sites as it was felt that this process was not in keeping with the ethos of a pragmatic “real‐world” trial.

### 2.3 Analysis of implementation challenges

In order to provide a systematic means of summarizing key challenges to implementation and focus our discussion on ways to mitigate potential difficulties in running a pragmatic trial, we carried out a thematic analysis of data from supervision records. Supervision records were word files that documented the content of meetings between members of the research team responsible for overseeing implementation and the site supervisors, as well as email correspondence between the research team and supervisors. These records and email correspondence were maintained by the research team. We carried out the thematic analysis of anonymized versions of this data in line with the steps outlined by Clarke and Braun (2013). The first step involved the first author (KB) reading anonymized versions of the logs and email correspondence several times to facilitate familiarization and highlight relevant issues. Next, a more detailed analysis was carried out to identify initial codes using an inductive, data‐driven approach. Related codes were grouped into “code families.” Data for each code and the relationships between codes was explored by KB and in conjunction with an undergraduate researcher. This enabled codes to be grouped together to form overarching themes, which were verified and refined as the analysis proceeded. Reflexivity is the process of acknowledging and reflecting upon the researcher's role and reflective experience (Clarke & Braun, 2013). KB is a clinical psychologist with experience in the delivery and supervision of a number of different trials of psychosocial interventions. The other authors who commented on the analysis are all experienced trialists of psychosocial interventions.

## 3 RESULTS AND DISCUSSION

There were six main themes in the data, which relate to implementation challenges. We use these themes to structure our discussion of how to successfully overcome challenges and run a pragmatic trial of a complex intervention. In doing so, we draw on both our past experience of running trials (foreseen problems) and lessons learnt in the current study (unforeseen problems).

### 3.1 Staff absences and leaving posts

One of the most significant challenges to implementation that arose during supervision sessions with the research team was staff absences and turnover. Difficulties in identifying and retaining people to deliver interventions is a secondary recruitment target, which is a usually overlooked but common problem for trials of complex interventions (Biggs et al., 2020) and can reflect the frequent high levels of staff sickness and turnover within health services (Willard‐Grace et al, 2019). The issue affected both facilitators and supervisors but did not disrupt the delivery of the intervention due to a number of measures we put in place. First, we encouraged sites to train more than the two required facilitators to deliver the intervention before they commenced delivery. In this regard, we also recommended that additional facilitators were introduced to group participations at the outset in the event that they needed to step in. There is a risk that these facilitators may forget aspects of the training if they are not delivering the intervention on a weekly basis, but this was less of a concern given that the group was co‐facilitated. Second, we delivered bespoke training to new facilitators or supervisors, which was supported by online resources. We used this system when people left a post or to train extra staff at sites where intervention or supervision delivery were proving difficult. Third, following queries from sites about what to do in the case of staff absences, we had a clear protocol for sites to follow in the event of a planned or unplanned absence, which involved liaising closely with the research team to ensure that sessions went ahead unless totally unavoidable and that the most suitable cover was provided. If no trained facilitators were available, substitute facilitators included supervisors or other clinically experienced staff supported by the research team through phone calls.

### 3.2 Participant lack of engagement with intervention

Another implementation issue that was discussed within supervision with the research team was that of participants not attending group intervention sessions. We sought to recruit 12–13 people per group, anticipating an average weekly attendance of 6–8 participants. Despite low numbers at some sessions (defined as less than four participants), we made a post hoc decision to go head and run groups with less than four people. We considered that it was more ethical to run the session for those who attended than to cancel it. When group sessions regularly had low numbers, supervisors noted that this impacted upon the morale of facilitators and we encouraged supervisors to reflect on these reactions as part of supervision. Reasons for participant nonattendance identified by supervisors included holidays, engagement in other social activities, hospital appointments, illness, and problems with travel. The former two reasons probably reflected the fact that participants in our trial were relatively high functioning despite their diagnosis (Wright et al., 2019). Conflicts with hospital appointments were due in part to our population being older and therefore more likely to be experiencing comorbid health problems. The challenges with travel were somewhat foreseen and resulted from the fact that some people were no longer able to drive, lacked confidence in using public transport (which could also be unreliable with limited coverage in some locations) and carer reluctance to let participants travel independently. In a number of instances, facilitators were encouraged through supervision to work with people to overcome travel challenges (e.g., practicing the route, sharing lifts, etc.). However, such problem solving needed to be discussed before the individual attended the first group meeting and we advised facilitators that the first one‐to‐one session should be used for this purpose. Notifying people in advance of the dates and times of each session also helped to reduce clashes of different commitments. The fact that the intervention was delivered over 16 sessions (12 group and 4 individual) helped to ensure that even if people could not attend every session they would still be able to receive a significant therapeutic dose (defined as attending 10 of the available 16 sessions including the one‐to‐one sessions).

### 3.3 Supervision delivery

Problems in the delivery of supervision due to lack of perceived need by facilitators or the busy schedules of supervisors were raised as issues by supervisors in monthly meetings with the research team. We anticipated attendance at supervision may have been an issue, which was a primary reason for recording and monitoring supervision using logs, which were submitted to the research team. Staff level of engagement in supervision often reflects the organization's culture and whether or not it is supportive of supervision (Snowdon et al., 2020). Where these problems occurred, we discussed possible solutions with the supervisors themselves or site principal investigators. Factors that facilitated the delivery of supervision sessions, which were ideas generated with supervisors during the trial, included delivering some supervision sessions via online platforms when supervisors and facilitators were based at different sites, scheduling all supervision sessions in the diary in advance of intervention delivery commencing and training a second supervisor if one supervisor was feeling overburdened. The commitment of service managers to deliver interventions and the supervision structure to support it is also key and involving these individuals during the design or set up phase helped to ensure this commitment as highlighted in previous studies (Raphael et al., 2021).

### 3.4 Group dynamics

The dynamics between participants during group sessions and how this could affect delivery of the intervention were issues that were often raised and addressed within supervision and also raised with the research team by supervisors. The impact of group dynamics on delivery of group‐based interventions has been previously documented in trials of group‐based interventions (Biggs et al., 2020), but also in routine clinical settings (Montgomery, 2002). Although there were no easy solutions to the problems posed, we recognized that it was important for facilitators to have an opportunity to reflect upon the difficulties they were facing during supervision and collectively think of ways to manage the situation, particularly as supervisors reported that these difficulties during the trial could negatively impact upon the enthusiasm of facilitators. A commonly reported challenge that our supervisors identified was that of group members being passive, making it hard for facilitators to engage them in collaborative decisions about how the group should be run and which topic areas to cover. Some supervisors reported to the research team that this was a particular problem for less experienced facilitators who were inclined to make decisions on behalf of the group. However, according to supervisors, some groups and facilitators did grow in confidence over time, resulting in more collaborative decision making by participants. This increase in confidence was attributed to greater experience in running the groups and suggests that more opportunities to role‐play difficult group dynamics within training or opportunities to initially co‐facilitate with more experienced staff may have been beneficial. Consistent with previous research, some supervisors highlighted a mismatch between the group of people who would be picked as ideal candidates for each group versus those consecutive people meeting study inclusion criteria who had been randomized to the intervention arm of the trial (Biggs et al., 2020). Supervision was used to discuss differences between delivering interventions as part of a research trial and delivering a group intervention in clinical practice.

### 3.5 Time to deliver the intervention

Facilitator workload and time pressures was a frequent theme raised during supervision. This barrier was anticipated and not surprising given how overstretched health services often are (Willard‐Grace et al., 2019). In order to minimize the impact of these constraints, we tried to ensure that the facilitators had ring‐fenced time to deliver the intervention through clear agreements with their employing organizations and by asking the supervisors to regularly check that people were being released from other duties for the agreed periods of time. These regular checks ensured that the research team could be alerted to problems early on and highlight any problems with site principal investigators who had accepted overall responsibility to ensure the delivery of the intervention. Supervisors also frequently worked with facilitators to help them manage their time and assert themselves if asked to carry out competing demands during time that had been allocated to the study. A number of sites did, however, reflect that preparing for sessions took longer than anticipated, especially during the early stages of the study when they were less familiar with the intervention. This highlights the importance of ensuring staff build in sufficient preparation time early on. Although we had previously piloted the intervention delivery (Sprange et al., 2015), this had involved more highly trained facilitators who may have been able to able deliver the intervention more efficiently. Delivering an intervention with less experienced staff may reduce the costs of the intervention and may increase the availability of potential supervisors, but the fact that less experienced staff may require more time and support to get the intervention up and running must be factored into planning.

### 3.6 Ethos of intervention and way it is delivered

The ethos of the intervention focused on working in partnership with people with dementia, enabling them to make their own decisions within the sessions and their own lives. The pre‐intervention training placed a strong emphasis upon the importance of challenging paternalistic attitudes and risk aversion, which are common within the health service (Shapiro, 2010). Nonetheless, conflict between the ethos of the intervention and risk averse culture of the host organizations frequently emerged throughout intervention delivery. This issue was particularly apparent in relation to the out‐of‐venue activities, which were a key aspect of the intervention. Out‐of‐venue sessions were to help people maintain independence and practice new or neglected life skills in community settings. Supervisors reported that facilitators often raised concerns about risks associated with these activities during supervision. In some instances, such concerns had resulted in facilitators wanting to avoid community‐based activities altogether. It was therefore important to address this issue by regularly checking with supervisors regarding the scheduling of out‐of‐venue activities and encouraging them to manage facilitator anxieties as part of supervision. For example, supervisors were encouraged to help facilitators to articulate specific concerns about what might go wrong during community‐based activities and think through contingency plans in event of their fears be realized. Part of this process also involved reminding supervisors of conveying the benefits of positive risk taking and the need to respect participants’ choices if they had capacity to make the decisions in question.

Two further issues arose during the trial, which were related to the delivery of the intervention. First, facilitators were required to write summaries at the end of each session, which could then be used as an aide memoir by participants. The research team regularly checked these summaries for two reasons, first to review the content of the intervention being delivered and second to determine the accessibility of such records for participants. This process was also very important in alerting us to the use of clinical language by some facilitators. Supervisors were subsequently asked to review end‐of‐session summaries as part of supervision and provide facilitators with guidance on how to use lay language and dementia friendly presentation, with some improvements noted over time.

Second, supervisors reflected that those facilitators that were less experienced often lacked confidence in delivering the one‐to‐one sessions possibly due to the lack of specific detail about what to cover within the intervention manual and training. When we reviewed the facilitators’ records of the sessions, we also noted that one‐to‐one sessions were sometimes just a recap of the previous group sessions with the individual. In response to these issues, we asked supervisors to encourage facilitators to think in more detail about how to tailor one‐to‐one sessions to the needs and wants of each person. We proactively encouraged supervisors to feedback both strengths and possible areas for improvement to facilitators, with the knowledge that supervisors can sometimes be less able to provide feedback in relation to the latter (Lefroy et al., 2015).

## 4 CONCLUSIONS AND SUMMARY OF RECOMMENDATIONS

This paper summarizes challenges that arose in implementing and evaluating a psychosocial intervention for people with mild dementia as part of a complex RCT. It also outlines how we used our collective experience from both previous studies and within this current study to circumvent and deal with these challenges. It is important to identify and surmount such challenges in order to uphold the validity of the trial findings and provide value for money (Tosh et al., 2011). Key challenges such as staff attrition, varying levels of participant and staff participation in aspects of the intervention and lack of adherence to the ethos or content of the intervention are likely to be faced by other researchers who seek to implement and evaluate complex interventions in real world settings. We hope that some of the strategies we used to circumvent these challenges may be useful for others. Other challenges and potential solutions are more specific to the nature of our intervention and our target participants. We believe that this paper is particularly timely given the recent proliferation of psychosocial interventions for people with a diagnosis of dementia, as well as for other groups of people living with complex long‐term conditions.

One of the most significant challenges to implementation that we successfully circumvented by training additional facilitators and supervisors was staff absences and turnover. We therefore recommend that implementers should build in sufficient resource to train additional staff and offer this training in a range of formats on a regular basis. There should also be protocols in place for introducing potential new staff to participants and clear protocols regarding the skills and qualities of those who might be substituted. In terms of maximizing participant attendance at group sessions in particular, we would recommend slightly over recruiting to groups to ensure the number of participants per group session does not drop to unacceptably low levels for either facilitators or the participants themselves. Where attendance is low, we also recommend that facilitators work closely with individual participants to identify and solve problems as far as is possible. This solution‐focused thinking should be built into the early stages of the intervention, including the possibility of scheduling all intervention sessions in advance. We would also recommend using supervision to help facilitators reflect upon issues that low attendance may raise for them in terms of morale. To maximize staff attendance at supervision (which is an essential mechanism for ensuring effective delivery of interventions), we recommend a means of regularly monitoring levels of supervision and addressing local barriers, which could be both attitudinal and pragmatic, in a timely manner.

Within the context of a trial of an intervention involving delivery of group sessions, there may be little that can be done to circumvent the inherent challenges with group composition, unless recruitment rates are high and groups are running simultaneously and in close locations. However, it is possible to reduce the impact of resulting difficulties in group dynamics by using supervision to raise, validate and, where possible, address problems. Recruiting facilitators experienced in group work and/or have good skills engaging others may also help address staff lack of confidence, although this may not always be possible due to the aforementioned difficulty in recruiting intervention deliverers.

In line with other trials and research exploring barriers to implementing interventions in routine practice (Raphael et al., 2021), we found that it is essential that staff are given ring‐fenced time to deliver the interventions and that this is supported by senior and immediate level management. We also recommend that the time needed for novice facilitators to deliver the intervention and the consequent need for adequate backfill should not be underestimated to avoid building resentment from either intervention deliverers or their managers if the work is more time‐consuming than expected.

Reasons for lack of adherence to the ethos of the intervention or its content were specific to the intervention, which we were implementing and evaluating. For example, our findings highlighted the some staff within community services for people with dementia may not have been fully socialized or equipped to work in “dementia friendly” ways. Nonetheless, issues of facilitator adherence are pertinent across all trials and are likely to be more significant in implementing complex interventions in the context of pragmatic trials or routine clinical practice (Tosh et al., 2011). Our findings highlight the importance of providing adequate training on all aspects of the intervention, clear and detailed manuals, and monitoring of adherence within the context of supervision with oversight from experienced clinicians within the research team. Within pragmatic trials, there is, however, a careful balancing act between implementing the intervention as intended without enhancing the intervention beyond what would ever be possible to deliver in real world settings. One way to manage this balancing act is for researchers to co‐create interventions with key stakeholders such as potential participants, their carers, intervention deliverers, and their managers (Richard et al., 2017).

## AUTHOR CONTRIBUTIONS

Conceptualization: KB, GM, KS, and CC. Formal analysis: KB. Funding acquisition: GM and CC. Writing—review and editing: all authors. Writing—original draft: KB.

## CONFLICT OF INTEREST

None to declare.

## TRANSPARENCY STATEMENT

The manuscript is an honest, accurate, and transparent account of the study being reported.

## ETHICAL APPROVAL AND CONSENT TO PARTICIPATE

This paper is a commentary so did not receive ethical approval per se but the trial we refer to had full ethical approval and participant written informed consent. The trial protocol was approved by UK Leeds East Research Ethics Committee, on 01/07/16, reference number 16/YH/0238.

## FUNDING

UK NIHR Health Technology Assessment Programme (14/140/80).

## CONSENT FOR PUBLICATION

We gained consent to publish this paper from all supervisors within the trial.

### PEER REVIEW

The peer review history for this article is available at https://publons.com/publon/10.1002/brb3.2436

## Data Availability

Anonymized data that support the findings of this study are available on request from the corresponding author. The data are not publicly available due to privacy or ethical restrictions.
